# Supplementary material for: Patterns of Intron Gain and Loss in Fungi
Source: PLoS Biol. 2004 Nov 30;2(12):e422. doi: 10.1371/journal.pbio.0020422 (PMC532390; doi:10.1371/journal.pbio.0020422)
Supplement: Table S1 — Also available at http://genes.mit.edu/NielsenEtAl/. (4.3 MB ZIP). [file pbio.0020422.st001.zip › NielsenEtAl/html/850.html]

AN1015.1.NCU07027.1.MG01819.1.FG09613.1


```
 CLUSTAL W (1.82) Multiple Sequence Alignments - Introns Inserted


Sequence 1: NCU07027.1	905 aa
Sequence 2: FG09613.1	887 aa
Sequence 3: MG01819.1	998 aa
Sequence 4: AN1015.1	822 aa
Alignment Length: 1019 aa
Number Identitical Residues: 560 aa
Alignment Score (without introns) 26012


MG01819.1 	MATSLALNVTLCFTATFPTEKLDELEQNCAWSLDSGTGSCTEPR0YCRARKRIAA2LMAK
NCU07027.1	--------------------------------------------~----------~----
FG09613.1 	--------------------------------------------~----------~----
AN1015.1  	--------------------------------------------~----------~----
          	                                                            

MG01819.1 	RVGEAETLFQ0LCLVSQHSSGAPPPTGLIIPQLPLAIDR2GILPHDLRPVAQDKMATDKP
NCU07027.1	----------~----------------------------~--------------MASNTT
FG09613.1 	----------~----------------------------~--------------MATEQ-
AN1015.1  	----------~----------------------------~--------------------
          	                                                            

MG01819.1 	QRIPMRERRPSSGAPLVDITGSVSPAGVSRPKHKRTLTGFGAQEIKSVEA1SIPEGQRAA
NCU07027.1	QRVPLRERRPSVGAPLVDIQGGVAPAGVSRPKHKRTLTGFGPGEIKNVEA1SIPEPQRKA
FG09613.1 	QRLPTRERRPSTSAPIVDIQGAVGPTGISRPKHTRTATGFGPSEIKNFEA1SIPEPQREA
AN1015.1  	-----------MSAACMLIQ----PRDLCSQKH-----------------~---------
          	            .*. : *     * .:.  **                           

MG01819.1 	WSKHQAKPFQDKEEFENEVVRHIETTLARSMFNCNES~AAYSAAGLAFRDRLIMEWNKTQ
NCU07027.1	WLAHQTSGFKDKDGFETEVVRHVETTLARSMYNCDEQ~AAYSACSLAFRDRLILEWNRTQ
FG09613.1 	WKRNQSRGFTGKDGFEQEVVRHVETTLARSVLNCDEN~AAYAATSLAFRDRLILDWNRTQ
AN1015.1  	-----------------ELVRHVETTLARSLYNCDEL2AAYSGTALAFRDRLIIEWNKTQ
          	                 *:***:*******: **:*  ***:. .********::**:**

MG01819.1 	QRQTFEDKKRVYY~LSLEFLMGRTLDNAMLNLNLKDVAKQ1GLSELGFNVEDIIGEERDA
NCU07027.1	QRQTFADSKRVYY~LSLEFLMGRALDNAMLNIGQKDVAKA~GLAELGFRIEDVIEQEHDA
FG09613.1 	QRQTYRDSKRVYY~FSLEFLMGRALDNAMLNVGQKDIAKA1GLSELGFRIEDIITQENDA
AN1015.1  	QRQTLNDQKRVYY1LSLEFLMGRTLDNAMLNVGMKDVARE1GLSDLGFRIEDVVSQEHDA
          	****  *.***** :********:*******:. **:*:  **::***.:**:: :*.**

MG01819.1 	ALGNGGLGRLAACFLDSLASLNYPAWGYGLRYRYGIFKQEIVDGYQVEVPDYWLDQNLWE
NCU07027.1	ALGNGGLGRLAACFLDSLASLNYSAWGYGLRYRYGIFKQEIIDGYQVEVPDYWLDFNPWE
FG09613.1 	ALGNGGLGRLAACFLDSLASLNYPAWGYGLRYRYGIFKQEIVDGYQVEVPDYWLDFNPWE
AN1015.1  	ALGNGGLGRLAACLLDSLATLNYPAWGYGLRYRYGIFKQEIVDGYQVEIPDYWLDFNPWE
          	*************:*****:***.*****************:******:****** * **

MG01819.1 	FPRHDVTVD~--IQFYGHVEKSQESSGSKTSANWVGGETVTAIAYDMPIPGYATPTTNNL
NCU07027.1	FPRHDVTVD~--IQFYGHVTKRTDDNG-KTIATWEGGEIVKAVAYDVPIPGYATPSTNNL
FG09613.1 	FPRHDVVVD0EQIQFFGHVRKTTDSNG-KSVAIWEGGEIVQAVAYDVPIPGYDTPTTNNL
AN1015.1  	FPRHEITVD~--IQFYGWVRTYEDENG-KTVHSWQDGETVQAVAYDVPIPGYGTRTTNNL
          	****::.**   ***:* * .  :..* *:   * .** * *:***:***** * :****

MG01819.1 	RLWSSKAASGEFDFQKFNSGEYESSVADQQRAETISAVLYPNDNLERGKELRLKQQYFWV
NCU07027.1	RLWSSKAASGEFDFQKFNSGDYENSVADQQRAETISAVLYPNDNLDRGKELRLKQQYFWV
FG09613.1 	RLWSSKASGGEFDFQKFNNGDYESSVADQQRAETISAVLYPNDNLDRGKELRLKQQYFWV
AN1015.1  	RLWSSKAASGEFDFQKFNAGDYESAVADQQRAETISAVLYPNDNLDRGKELRLKQQYFWC
          	*******:.********* *:**.:********************:************* 

MG01819.1 	AASLYDIVRRFKKSKRAWREFPEQ0VAIQLNDTHPTLAVVELQRILIDLEGLDWDDAWNI
NCU07027.1	AASLYDIVRRFKKSRRPWKEFPDQ~VAIQLNDTHPTLAVVELQRILVDLEGLDWEEAWNI
FG09613.1 	AASLYDIVRRFKKSSRPWREFPDQ~VAIQLNDTHPTLAIVELQRILIDIEHLEWDLAWDI
AN1015.1  	AASLHDIVRRFKKTGRPWSEFSDQ~VAIQLNDTHPTLAIVELHRILIDIEGLDWDVSWEI
          	****:********: *.* **.:* *************:***:***:*:* *:*: :*:*

MG01819.1 	VQST~FGYTNHTVLPEALEKWPVGLIQHLLPRHLQIIYDINLFFLQSVERQFPGDRDLLS
NCU07027.1	VTNT~FGYTNHTVLPEALEKWSVPLFQHLLPRHLQLIYDINLFFLQSVERKFPKDREMLA
FG09613.1 	VVKT0FSYTNHTVLPEALEKWPVGLIQHLLPRHLQIIYDINLFFLQKVEKAFPNDRDILS
AN1015.1  	VTNT~FGYTNHTVLPEALEKWSVPLLQNLLPRHMQLIFEINLYFLQFVEKKFPDDREILS
          	* .* *.**************.* *:*:*****:*:*::***:*** **: ** **::*:

MG01819.1 	RVSIIEEGQTKMVRMAHLAIIGSHKVNGVAELHSDLIKTTIFRDFVEIFGPDKFTNVTNG
NCU07027.1	RVSIIEESQPKMVRMAHLAIVGSHKVNGVAELHSDLIKTTIFKDFVEVFGPDKFTNVTNG
FG09613.1 	RVSIIEESQTKMVRMAFLAIVGSHKVNGVAELHSDLIKTTIFKDFVEIYGPDKFINVTNG
AN1015.1  	RVSIIEESHPKMVRMAHVAVIGSHKVNGVAELHSDLIQSTIFRDFVAIYGPDKFTNVTNG
          	*******.:.******.:*::****************::***:*** ::***** *****

MG01819.1 	ITPRRWLHQANPKLSELISTKCGSYDFLKDLTGLNELEKWVKDEEFRKEWAAIKRSNKAR
NCU07027.1	ITPRRWLHQANPRLSELISSKTGSQNFLKDLTELAKIEHYKDDKAFRKEWAEIKYANKVR
FG09613.1 	ITPRRWLHQANPRLSELIASKVGGNGFLKDLTNLNQLEKYAEDKEFRKEWSEIKYANKVR
AN1015.1  	VTPRRWLHQANPRLSDLIASKLGGYDFLTDLTLLDQLEDYADDKDFQREWVEIKTSNKLR
          	:***********:**:**::* *. .**.*** * ::*.: .*: *::**  ** :** *

MG01819.1 	LADYIKRTTGVTVSPDALFDVQVKRIHEYKRQQMNIFGVIHRYLALKAMTPEEREKQLPR
NCU07027.1	LAKHIKKTTGVDVNPSALFDVQVKRIHEYKRQQMNIFGVIHRYLTLKSLSPEERKKFQPR
FG09613.1 	LAKLIKSLVGVTVNPSALFDVQVKRIHEYKRQQLNIFGVIHRYLYLKSLTPEERKKVVPR
AN1015.1  	LAKHIKETTGYSVNPNSLFDVQVKRIHEYKRQQLNIFGVINRYLKIKSMSGEEKKKVQPR
          	**. **  .*  *.*.:****************:******:*** :*::: **::*  **

MG01819.1 	VSIFGGKAAPGYWMAKQIIHLINSVGAVVNKDEDIGDKLKVVFLEDYNVSKAEMIIPAND
NCU07027.1	VSIFGGKAAPGYWMAKQIIHLINAVGAVVNNDKDIGDLLKVIFLEDYNVSKAEMIIPASD
FG09613.1 	VSIFGGKAAPGYWMAKQIIHLVNAVGSVVNNDEDIGDLLKVIFLPDYNVSKAEIITPASD
AN1015.1  	VSIFGGKAAPGYWMAKTIIHLINEVSVVVNNDPDVGDLLKVIFIEDYNVSKAEIICPASD
          	**************** ****:* *. ***:* *:** ***:*: ********:* **.*

MG01819.1 	LSEHISTAGTE~ASGTSNMKFVLNGGLIIGTCDGAN0------------------IEITR
NCU07027.1	LSEHISTAGTE2ASGTSNMKFVLNGGLIIGTCDGAN~VSNNPLFYAMSSLANPLQIEITR
FG09613.1 	LSEHISTAGTE2ASGTSNMKFVLNGGLIIGTCDGAN0------------------IEITR
AN1015.1  	ISEHISTAGTE~ASGTSNMKFVLNGGLIIGTCDGAN0------------------IEITR
          	:********** ************************                   *****

MG01819.1 	EIGENNIFLFGNLAEDVEDLRHAHTYGKTHAIDPELLKVFDAIQAGKFGEPQNFGSLIAA
NCU07027.1	EIGEQNIFLFGNLAEDVEDIRHNHTYG-SYTVDPDLVKVFEAIEKGTFGEPNDFMGMISA
FG09613.1 	EIGENNIFLFGNLAEDVEDLRHNHQYG-SHEIDPDLQKVFAEIEKGTFGSVHDFSALVAA
AN1015.1  	EIGEQNIFLFGNLAEDVEEIRHKHMYS-GFTLDPQLARVFDAIRSGTFGEASHYSALLAS
          	****:*************::** * *.  . :**:* :**  *. *.**.  .: .::::

MG01819.1 	IKDHGDYYLVSDDFSSYLDTHKLVDESYRDQEGWITKCITSVARMGFFTSDRCINEYAEE
NCU07027.1	VRDHGDFYLVSDDFHSYIETQELVDKAYRDQEGWITKSIESVARMGFFSSDRCINEYAEG
FG09613.1 	VRDHGDYYLVSDDFHSYNETHKLVDEAYQNQEEWIKKTITSVSRMGFFSSDRCIDEYAES
AN1015.1  	IAEHGDYYLVSDDFNSYIKTQEMVDEAFKDREEWVSKSITSVARMGFFSTDRVINEYAES
          	: :***:******* ** .*:::**::::::* *:.* * **:*****::** *:**** 

MG01819.1 	IWNIEPLKVGA-
NCU07027.1	IWNIEPLAVKDQ
FG09613.1 	IWNTEPLVIHD-
AN1015.1  	IWNIEPLPVRD-
          	*** *** :
```
